# Medical Extracts—Practical and Philosophical, No. II

**Published:** 1873-06

**Authors:** L. B. Anderson

**Affiliations:** Virginia


### MEDICAL EXTRACTS—PRACTICAL AND PHILOSOPHICAL—NO. II.

BY L. B ANDERSON, M D , OF VIRGINIA.

### *MEASLES.*

From the mountains to the sea this disease has prevailed in Virginia during the past few months. An epidemic influenza was prevailing at the time, and hence the almost universal pulmonary complications. In some localities, either from endemic influence or improper treatment, it has been very fatal—one physician having lost a dozen patients in a few weeks. I design to make a few practical suggestions prompted by the experience of the last few weeks.

When there is evident bilious derangement, calomel in full doses, combined with castor-oil, is a valuable initiative. When the disease has progressed, when given in small doses it is apt to produce an obstinate diarrhœa.

Blisters are decidedly objectionable at all times and stages, until several days after the eruption has disappeared, when they prove beneficial in bronchial and pneumonic complications.

Heating and stimulating beverages, so generally used by the vulgar, sometimes promote the eruption and hasten its maturation and the convalescence of the patient, but is a hazardous practice; for, when the centripetal action is too potent to be overcome by them, they invariably increase its energy, and endanger the life of the patient. When there is no local disease, no diarrhœa, no constipation, no cough, and the vital functions are in proper exercise, no treatment is needed. A room and bed comfortably warm; warm, nutritious food; shaded windows, and perfect repose, will meet every indication. But there are few cases in which the onset of the morbid action does not produce more or less torpor of liver and kidneys, heat and dryness of the skin, constipation of bowels, and congestion of the pulmonary vesicles—thus obstructing all the channels of depuration and vivification to a greater or less degree. It is, therefore, generally of the first importance that medical aid should be early and prudently afforded. The indications are plain: 1. To rouse the dormant energies of the vital organs, promote the secretions from the liver, kidneys and skin, and empty the loaded pulmonary capillaries. This is, happily, generally attained by a full emetic of ipecac wine. 2. To promote and preserve these secretions and functions after the action of the emetic, give a full dose of calomel and oil; apply hot mush poultices over the chest, and give small and frequently-repeated doses of wine of ipecac and spirits nitre.

When bronchial and pulmonary complications are developed, cupping often—dry or wet—over the chest, according to the requirements of the pulse; epithems of kerosene-oil, covered with a flannel pad, and antimonial wine, are highly beneficial. Where the larynx is principally or concomitantly affected, tr. sanguinaria from the recent root, and chlorate of potass., with gargles of carbolic acid, and kerosene oil and flannel to the neck, are indicated. If there are serious abdominal complications, the above epithems and poultices, persistently used, with ipecac internally, and dry cupping over the affected region, will generally meet the end required. These general remarks will indicate the treatment which has resulted favorably in every case I have treated this season—embracing a large number, with all the complications mentioned, and every variety of complication and predisposition.

Hot-water tea, made of equal parts of cream and boiling water, slightly sweetened, constitutes the sole article of diet; sassafras, elm or gum mucilage the only beverage. When the symptoms are decidedly asthenic, after removing congestions, subduing inflammations, opening the bowels, and promoting the secretion from the kidneys by the means indicated, I do not hesitate to give wine cautiously. The great work of the physician is to guard well the weak points of the citadel entrusted to his care. Measles always directs its concentrated energies to such points. Study well the topography, if you please, of the system; ascertain the most vulnerable points, and, waiting not for the onset to be made, construct your barricades, and by every stratagem decoy the enemy from those points. By thus acting, you will be able easily to manage what otherwise would speedily be a hopeless case.

